# Effectiveness of the digital parental-family-centered program for problematic gaming and screen use in a clinical child and adolescent population

**DOI:** 10.1016/j.puhip.2026.100848

**Published:** 2026-08-24

**Authors:** Emma Claesdotter-Knutsson, Sophie Wilts, Marie Werner, Frida André, Mitchell J. Andersson

**Affiliations:** a Department of Clinical Sciences, Faculty of Medicine, Lund University, Lund, Sweden; b Child and Adolescent Psychiatry, Regional Outpatient Care, Skåne University Hospital, Lund, Region Skåne, Sweden; c Faculty of Medicine, University of Amsterdam, Amsterdam, the Netherlands; d Primary Health Care, Region Dalarna, Sweden

**Keywords:** Digital intervention, Problematic gaming, Problematic screen use, Family intervention

## Abstract

**Objectives:**

Problematic gaming and screen use may be associated with psychosocial, academic, and mental-health difficulties in children. The digital Parental-Family-Centered Program (P-FAME) is a brief, digitally delivered, parent-focused program developed in Child and Adolescent Mental Health Services. This study examined its feasibility and preliminary associations with change in child screen time, family functioning, and parental stress over time.

**Study design:**

Prospective single-group pre–post feasibility study.

**Methods:**

Twenty-nine parents of 22 children (mean child age 10.6 years) participated in three online group sessions delivered over five weeks. Standardized measures were administered at baseline and post-intervention, including Screens-Q (screen time and rules), SCORE-15 (family functioning), and the Parental Stress Scale (PSS). Linear mixed-effects models accounting for clustering within families estimated change from pre-intervention to post-intervention.

**Results:**

From baseline to post-intervention, children's weekly screen time decreased (adjusted mean difference: −1400 min/week, *p* < .001; *d* = −1.81), as did parental stress (*p* = .036; *d* = −0.63). The PSS stress subscale also decreased (p = .048, *d* = 0.59). No statistically significant changes were observed in overall family functioning or parental satisfaction. Child ADHD diagnosis and age did not significantly moderate the observed changes.

**Conclusions:**

In this uncontrolled clinical study, participation in P-FAME was associated with lower child screen time and parental stress. The findings provide preliminary feasibility and outcome signals that require confirmation in adequately powered controlled studies with longer follow-up and broader implementation measures.

**What this study adds**

This study provides preliminary evidence that participation in a brief, digitally delivered, parent-focused intervention within Child and Adolescent Mental Health Services was associated with lower child weekly screen time and parental stress in a clinically referred population.

The observed pattern is consistent with the possibility that parental awareness, reflection, and environmental structuring may be relevant targets for problematic screen use; however, the uncontrolled design does not establish mechanisms of change.

Session attendance and completion indicators suggest that the online group format was feasible for participating families in routine outpatient care; its scalability and acceptability require more comprehensive implementation evaluation.

**Implications for policy and practice**

The preliminary findings support further controlled evaluation of brief, online, parent-centered programs within Child and Adolescent Mental Health Services as a potential low-threshold response to problematic screen use.

Future policy and service development should consider parental competence, stress management, and relational strategies alongside child-directed regulation and screen time limits; the relative contribution of these elements should be evaluated empirically.

Future research should assess P-FAME's transferability to school health services, primary care, social services, and preventive settings.

## 1 Introduction

Most children in Sweden use the internet daily, with a substantial proportion exceeding the screen time limits recommended by the Swedish Public Health Agency. [1,2] Indeed, a recent report estimated that approximately 94% of Swedish children aged 8-12 years old play online games every day. [3] While digital media and gaming may confer certain benefits, heavy engagement carries notable mental health risks. Problematic screen use refers to poorly regulated digital-media use that is associated with functional impairment or negative consequences in daily life, such as difficulties at school, in social relationships, or in psychological well-being. [4] Meanwhile, excessive screen use (ESU) is used more narrowly to describe a high amount of screen use and may, but need not, be problematic. These behavioral patterns have been associated with various psychosocial issues including inattention, poorer academic performance, depression, anxiety, stress, and sleep disturbance. [[5], [6], [7], [8], [9], [10], [11], [12]] Beyond general patterns of problematic screen use and ESU, gaming disorder (GD) stands as a distinct diagnosis in the International Classification of Diagnoses (ICD-11) characterized by impaired control over gaming, increasing priority given to gaming, and continuation or escalation despite negative consequences. [13] Taken together, problematic screen use and related difficulties represent a growing public health concern with implications for children's mental health and development.

Children's development is shaped through interactions with their surrounding environment, particularly the family ecosystem. [14] Accordingly, the emergence, prevention, and management of ESU and problematic screen use may be influenced by familial factors. [15,16] Problematic screen use is increasingly understood as embedded in relational and family processes, [17] yet intervention research has largely targeted children and adolescents as the primary recipients of treatment. [18] This emphasis may overlook how parental responses, emotional well-being, and everyday interaction patterns contribute to the maintenance of, and potential responses to, screen-related difficulties. Family-focused approaches may therefore be relevant alongside strategies that strengthen parental coping, communication, and family functioning. [19,20] Qualitative studies of family-centered programs highlight parents' need for practical guidance and structured support when managing their child's screen use. [[21], [22], [23]]

These considerations may be especially relevant during middle childhood, when executive functions—including inhibition, working memory, cognitive flexibility, emotion regulation, and behavioral self-control—are still developing. Childhood is a potentially age-sensitive period during which behavioral and environmental experiences may influence executive-function-related outcomes. [24] Thus, considering child age and developmental stage when designing and evaluating parent-mediated interventions for screen-related difficulties is imperative.

The documented links between problematic gaming, ESU, and psychosocial difficulties underscore the need for accessible preventive interventions. Addressing difficulties at an early stage may reduce the risk of symptom escalation and secondary relational conflict. Low-threshold, parent-focused programs are particularly well suited to this need, as they can be integrated into routine services. Grounded in learning theory, emotion theory, and systemic developmental perspectives, [14,25] the Parental–Family-Centered Program for problematic gaming and excessive screen use (P-FAME) integrates evidence-informed components that target behavioral and relational processes. It combines psychoeducation, structured homework assignments, support for generalization to everyday contexts, and peer support through brief lectures, guided exercises, and therapist-led parent discussions. The program is intended to help parents identify behavioral functions and conflict-triggering factors related to screen use and to reflect on possible strategies for everyday routines. P-FAME is intended to serve as an early, digitally accessible and deliverable program to parents who experience their child's screen use as problematic.

This study aimed to evaluate (1) the feasibility of administering P-FAME to families affected by ESU or related difficulties in a Child and Adolescent Mental Health Services (CAMHS) outpatient setting, and (2) to preliminarily estimate associated changes in relevant parent and child outcomes. Leveraging both parent and child reports, we hypothesized that the intervention would be associated with decreased child screen time and parental stress as well as improved family functioning from baseline to post-intervention. We also explored whether child ADHD diagnosis or age was associated with differences in observed pre–post change.

## 2 Methods

### 2.1 Study design and participants

A prospective single-group pre–post feasibility design was used to examine change from baseline to post-intervention. Three cohorts of families participated; parents received the intervention, while children completed accompanying outcome measures. Cohort 1 participated from 30 April to 28 May 2025, cohort 2 from 21 May to 18 June 2025, and cohort 3 from 24 October to 21 November 2025. Available feasibility information comprised enrolment, session attendance, and analyzable questionnaire data.

Participants were recruited from CAMHS outpatient clinics in Skåne during spring and autumn 2025. Clinicians informed families reporting screen-related difficulties about the study and invited interested parents to participate. Enrolment followed CAMHS's standard digital routines, after which the principal investigator provided written and oral study information and obtained informed consent. Baseline and post-intervention questionnaires were administered two weeks before and two weeks after the intervention. Eligible participants were parents of children aged 6–12 years who reported family difficulties related to the child's screen use, spoke and understood Swedish, and had stable internet access and appropriate technical equipment. Families requiring an interpreter or lacking sufficient internet connection or technical resources were excluded.

### 2.2 Intervention (P-FAME)

The digital P-FAME is an early intervention for families in which at least one parent reports difficulties related to their child's screen use. The program comprises three 90-min online group sessions delivered over five weeks by two trained CAMHS Skåne counsellors. P-FAME draws on learning, emotion, and systems theory. [14,25] Across sessions, parents receive psychoeducation, complete structured home assignments, discuss generalization to daily family life, and participate in guided exercises and peer discussion. Session 1 provides psychoeducation on screen use, its possible consequences, and current recommendations, and introduces observation and reflection tasks for the home environment. Session 2 focuses on identifying the behavioral functions, maintaining factors, and conflict triggers that may be associated with the child's screen use. Session 3 addresses strategies to prevent and manage screen-related conflict, with emphasis on constructive family communication and the application of individualized strategies between sessions.

The intended targets are problematic screen-related behavior, family functioning, and parental stress. The two group leaders facilitated active participation through breakout rooms and counsellor-led discussions. The program did not contain a separate standardized contingency-management module, and it was not designed to provide a formal assessment of child compliance with screen-related rules.

### 2.3 Outcome measures

The primary participant-centered outcomes were change in weekly child screen time, family functioning scores, and parental stress scores. Secondary outcomes included change in screen use rules and perceptions of screen use, strengths, difficulties, and communication underpinning functioning within families, and stress and satisfaction of parenting. All parents and their respective children above the age of ten received questionnaires. Parents received the Screens Questionnaire [26] (Screens-Q), Systematic Clinical Outcome and Routine Evaluation [27] (SCORE-15), and Parental Stress Scale [28,29] (PSS), while the children above the age of ten years received the Screens-Q and SCORE-15. The SCORE-15 and PSS questionnaires were distributed digitally through *Amni Care*, a digital health platform used throughout Sweden. Screens-Q was not included in *Amni Care* and was thus sent to the families in paper format.

Child weekly screen time, screen-related rules, and perceptions of child screen use were assessed using Screens-Q, a standardized and validated measure of children's screen habits. [26] Screen time was estimated using six items, each measured on an eight-point ordinal scale ranging from “never” to “five or more hours,” differentiated by day type (weekday versus weekend) and modality (e.g., watching movies or playing games). Because most responses were provided in ranges (e.g., 1–29 min or 3–4 h), weekly screen time was estimated by summing category midpoints across modalities for weekdays and weekends, multiplying by five for weekdays and two for weekend days, and combining these totals. Parent-reported screen-related rules were measured using five binary items (0 = disagree, 1 = agree), with higher scores indicating endorsement of a greater number of rules. This score reflects the reported presence of rules rather than their consistency of enforcement, child compliance, or perceived effectiveness.

Change in self-reported family functioning was assessed using SCORE-15, a questionnaire consisting of fifteen items spanning three factors: family strengths, difficulties, and communication. Responses were rated on a five-point Likert scale, ranging from 1 (‘Describes us very well’) to 5 (‘does not describe us at all). [27] Scoring produces one cumulative score (Total SCORE-15) and three subfactors: *Total communication*, *Total strengths and adaptability*, and *Overwhelmed by difficulties.* Higher communication scores reflect poorer communication, whereas higher strengths scores reflect improvements in strengths and adaptability, and higher difficulties scores reflect fewer difficulties.

Change in parental stress was assessed using the PSS, [28,29] which comprises 18 items assessing common parenthood experiences and parenting-related stress across both positive and negative aspects. Responses are rated on a five-point Likert scale, ranging from 1 (Strongly disagree) to 5 (strongly agree). Scoring yields a total PSS-score, and two subscale scores: *Parental Stress*; and *Lack of Parental Satisfaction* (reversely scored).

Extant research demonstrates that these questionnaires exhibit satisfactory psychometric properties, supporting its use for repeated measurements in longitudinal research. [[26], [27], [28], [29]] Other relevant demographic data, including participants’ sex, the sex and age of their children, and the diagnosis serving as the basis for contact with the CAMHS, were also collected.

### 2.4 Statistical analysis

Descriptive statistics are summarized using means and standard deviations (*M*±*SD*) or medians alongside quartiles (*Mdn* [Q1-Q3]) where appropriate. Missing data were imputed for participants who completed at least 50% of the items within each scale. For screen time, missing items were imputed as zero. All remaining variables were imputed using the participant-level median of the respective scale.

Linear mixed-effects models were employed to estimate within-group change from baseline to post-intervention. Since each child and parent belonged to an overarching family cluster, nested models accounted for the hierarchical structure of the data. For the main analyses, timepoint (baseline versus post-intervention) and family-member type (child versus parent) were included as fixed effects. Random intercepts were specified for family groups and respondents nested within family groups. Exploratory models included interactions between timepoint and child ADHD diagnosis or age to examine whether these variables were associated with different observed changes over time. Sensitivity paired *t*-tests by family-member type were also conducted.

Model-based adjusted mean differences (*M*_*diff*_) alongside 95% confidence intervals and Cohen's *d* for paired data were calculated to quantify the unstandardized and standardized effects of the intervention on outcomes. Model assumptions pertaining to linearity, homoscedasticity, and normality of residuals were assessed through visual inspection of standardized residual and Q-Q plots. All analyses were performed in Rv4.4.1 [30] using *emmeans,* [31] *lme4* ,[32] and *performance* [33] packages.

## 3 Results

### 3.1 Demographics

A total of 29 parents of 22 children (*M*_*age*_ = 10.6 ± 1.5 years; range 8–13 years) participated in the intervention and were included in analyses (*N* = 51 individuals). Children younger than 10 years (*n* = 7) did not receive surveys because the survey battery was not intended for this age group; the final survey-respondent sample therefore comprised 44 individuals. Of the participating parents, 52% attended all three sessions (*M*_*sessions*_ = 2.48 ± 0.57). Missing item-level data were 7.1% for parents and 6.9% for children. These indicators describe participation among enrolled families; rates of eligibility, invitation, refusal, and post-intervention assessment completion could not be fully quantified because referral-pathway denominators were not prospectively recorded. Sample characteristics stratified by respondent type are presented in Table 1.

**Table 1:** Sample characteristics.

Characteristic	Family Member Type	
	Parents (*n* = 29)	Children (*n* = 22)
Cohort, *n* (%)		
1	10 (34.5%)	9 (40.9%)
2	11 (37.9%)	7 (31.8%)
3	8 (27.6%)	6 (27.3%)
Male sex, *n* (%)	6 (20.7%)	21 (95.5%)
ADHD dx, *n* (%)	—	13 (59.1%)
Age, *M* ± *SD*	—	10.59 ± 1.53

*Note.* ADHD = attention deficit hyperactivity disorder. dx = diagnosis.

### 3.2 Outcomes

For primary outcomes, child weekly screen time and parental stress were lower at post-intervention than at baseline after accounting for clustering within families and family-member differences (1400 min/week, *p* < .001; 2.4 points, *p* = .036, respectively). Overall, family functioning did not change significantly from baseline to post-intervention (*p* = .43). For secondary outcomes, the PSS stress subscale was lower at post-intervention (2.3 points, *p* = .048), whereas the remaining outcomes did not change significantly. Full descriptive statistics by timepoint and family-member type, together with model-based adjusted mean differences, are presented in Table 2.

**Table 2:** Primary and secondary outcome measures.

Primary outcomes	Family	Pre-intervention		Post-intervention		Intervention Effect	
	Member	*n*	*M* ± *SD*	*n*	*M* ± *SD*	Adj *M*_*diff*_ [95% CI]	*p*
Screentime (min/wk)	Child	14	3827 ± 1497	15	2625 ± 1227		
	Parent	27	3509 ± 1288	27	1936 ± 732	−1400 [-1745, −1055]	**<.001**
SCORE-15 (total)	Child	14	32.4 ± 8.2	12	32.2 ± 10.8		
	Parent	28	33.6 ± 8.9	24	33.2 ± 9.2	−0.9 [-3.0, 1.3]	0.43
PSS (total)	Child	—	—	—	—		
	Parent	28	63.0 ± 6.8	25	60.7 ± 9.9	−2.4 [-4.6, −0.2]	**.036**
**Secondary outcomes**							
*Screens-Q subscales*							
Rules	Child	14	2.14 ± 1.41	15	2.87 ± 1.81		
	Parent	27	3.74 ± 1.26	27	3.82 ± 1.39	0.32 [-0.16, 0.81]	0.19
*SCORE-15 subscales*							
Strengths	Child	14	9.7 ± 2.6	12	10.1 ± 3.9		
	Parent	28	10.9 ± 3.3	24	10.0 ± 2.9	−0.6 [-1.3, 0.2]	0.14
Difficulties	Child	14	18.4 ± 3.6	12	18.4 ± 4.6		
	Parent	28	16.9 ± 3.9	24	17.5 ± 4.4	0.4 [-0.6, 1.4]	0.44
Communication	Child	14	18.9 ± 3.3	12	19.4 ± 3.6		
	Parent	28	19.9 ± 3.2	24	19.4 ± 3.1	−0.1 [-1.1, 0.9]	0.83
*PSS subscales*							
Stress	Child	—	—	—	—		
	Parent	28	29.6 ± 6.8	25	27.3 ± 8.3	−2.3 [-4.6, −0.0]	**.048**
Satisfaction	Child	—	—	—	—		
	Parent	28	33.4 ± 5.3	25	33.4 ± 5.8	−0.0 [-1.1, 1.1]	0.93

*Note.* SCORE15 = Systematic Clinical Outcome and Routine Evaluation, PSS = Parental Stress Scale.

Summary statistics are unadjusted. Adjusted mean differences (Adj *M*_*diff*_) are based on linear mixed models averaging across family member types and accounting for clustering.

**Fig. 1:**
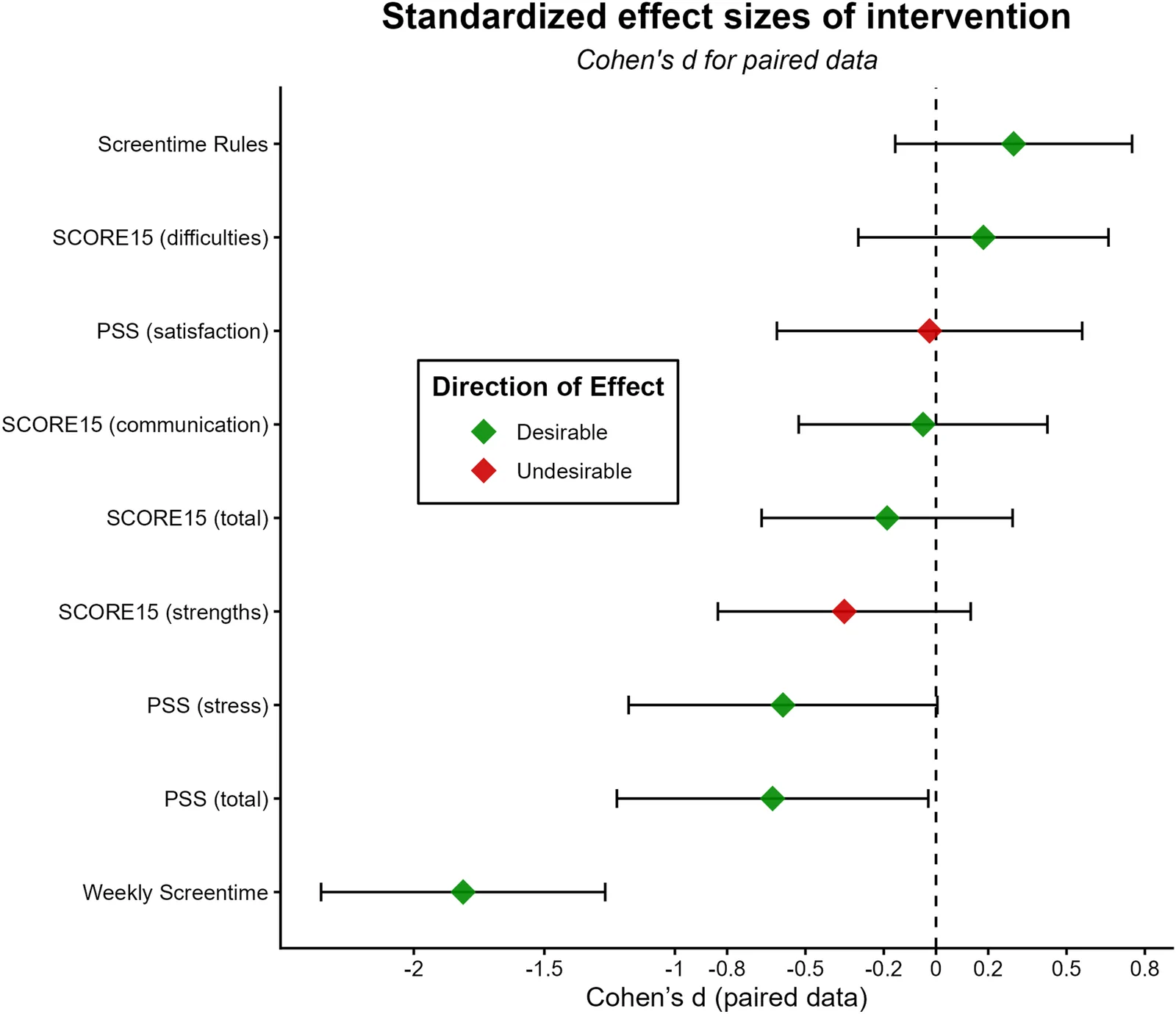
Standardized effect of intervention on study outcomes *Note*. SCORE15 = Systematic Clinical Outcome and Routine Evaluation, PSS = Parental Stress Scale. Effect sizes (*d*) presented alongside 95% confidence intervals.

Fig. 1 illustrates standardized pre–post change estimates (Cohen's *d* for paired data). The largest absolute standardized change was observed for child weekly screen time (*d* = −1.81), followed by parental stress and its stress subscale; smaller changes were observed for screen-related rules.

Child ADHD diagnosis and age did not significantly moderate the observed pre–post changes in the interaction models controlling for family-member type (*p*-values = 0.06–0.66). The age-by-timepoint interaction for family communication approached conventional statistical significance (*p* = .063), with a descriptive pattern of greater reported improvement in younger than older children (Table 3).

**Table 3:** Moderation of child ADHD and age on treatment outcomes.

Outcome	*ADHD Beta (DiD) [95% CI]*	*p*	*Age Beta (DiD) [95% CI]*	*p*
**Primary**				
Screentime (min/wk)	−18 [-709, 672]	0.96	53 [-187, 293]	0.66
SCORE-15 (total)	−0.71 [-5.03, 3.61]	0.74	−1.26 [-2.74, 0.21]	0.09
PSS (total)	−1.71 [-6.18, 2.76]	0.44	0.68 [-0.70, 2.07]	0.32
**Secondary**				
*Screens-Q subscales*				
Rules	−0.63 [-1.59, 0.32]	0.19	0.19 [-0.14, 0.53]	0.25
*SCORE-15 subscales*				
Strengths	0.15 [-1.36, 1.67]	0.84	−0.22 [-0.75, 0.32]	0.42
Difficulties	0.51 [-1.50, 2.52]	0.61	0.43 [-0.27, 1.13]	0.22
Communication	0.40 [-1.54, 2.34]	0.68	0.62 [-0.03, 1.27]	0.063
*PSS subscales*				
Stress	−0.91 [-5.60, 3.77]	0.69	0.91 [-0.51, 2.33]	0.20
Satisfaction	−0.84 [-3.07, 1.38]	0.44	−0.23 [-0.93, 0.47]	0.50

*Note*. DiD = difference-in-differences (i.e., interaction effect). SCORE15 = Systematic Clinical Outcome and Routine Evaluation, PSS = Parental Stress Scale. Pre-intervention and non-ADHD diagnosis served as reference groups. For example, a negative estimate for screen time indicates that children with ADHD had larger decreases in screen time than children without ADHD from pre-intervention to follow-up.

## 4 Discussion

We assessed the efficacy and feasibility of the P-FAME intervention for parents of children presenting with screen-related difficulties in a CAMHS setting. High attendance rates demonstrated strong intervention feasibility, and findings partially supported our primary outcome hypotheses: P-FAME was associated with reduced child screen time and parental stress, though no significant improvements were found in family functioning. Within a framework emphasizing parental mediation and behavioral change, the observed pattern is consistent with awareness- and support-based processes being relevant targets in a parent-focused program. However, the single-group pre–post design does not permit conclusions about mechanisms or establish that P-FAME caused the reduction in child screen time. The lack of statistically significant change in reported rules also indicates that changes in the presence of formal rules were not demonstrated in this sample. Future controlled studies should test whether parental reflection, environmental structuring, communication, or other hypothesized mechanisms account for subsequent changes in screen-related behavior.

The observed reduction in screen time is theoretically meaningful given reported associations between ESU and inattention, poorer academic performance, depression, anxiety, stress, and sleep disruption. [[5], [6], [7], [8], [9], [10], [11], [12]] Its clinical meaning nevertheless depends on what activities replaced screen time and whether changes are sustained. [6] The observed decrease in parental stress is compatible with the possibility that structured guidance, peer support, and practical tools are relevant to perceived parental control, but this interpretation remains tentative. Overall family functioning and parental satisfaction did not change significantly. A brief program may first influence concrete routines and parental stress, whereas broader relational functioning may require a longer duration, more intensive family work, or a longer period before change is detectable. It is also possible that the SCORE-15 was insufficiently sensitive to short-term change in this context. Parent–child differences in reported family strengths may additionally reflect differing temporal perspectives and relational processes rather than objective deterioration. [34]

The present findings are aligned with the broader interest in parent-directed approaches to children's screen use, [18] while remaining preliminary. Participation and session attendance provide initial feasibility information, but they do not by themselves establish acceptability, reach, implementation fidelity, or scalability. Broader research across digital interventions reveals substantial variation in intervention designs, engagement, adherence, and outcomes, [35] reinforcing the importance of evaluating implementation outcomes rather than assuming that a digital format is inherently scalable. Accordingly, P-FAME should be characterized as a promising low-threshold approach for further evaluation rather than an established scalable service model.

The exploratory age analyses should not be interpreted as evidence that developmental stage is unimportant. The absence of statistically significant moderation may reflect limited power, restricted age variation, or measurement limitations as well as a true absence of effect. Childhood has age-sensitive periods for executive-function-related outcomes [24]; therefore, age, executive functioning, and self-regulatory capacity remain clinically plausible candidates for tailoring parent-mediated screen-use interventions. Larger studies designed and powered for moderator analyses are needed before drawing conclusions about for whom P-FAME may be most helpful.

### 4.1 Strengths and limitations

This study has several limitations. The five-week intervention and two-week post-intervention assessment limit inference about durability and may have been insufficient to detect broader changes in family functioning. The small sample size limited precision and statistical power, especially for exploratory moderation analyses. The single-group pre–post design precludes causal attribution: regression to the mean, expectancy effects, concurrent treatment, seasonal variation, and natural changes in family routines cannot be ruled out. Recruitment and retention indicators were available for enrolled participants, but complete denominators for eligible, invited, declining, and post-intervention-assessed families were not prospectively recorded. This constrains evaluation of reach and retention. This limitation is particularly relevant given the impact of intervention design, adherence, monitoring, and participant characteristics on the outcomes of digital and home-based interventions. [35] Future P-FAME trials should incorporate more explicit implementation outcomes and test its efficacy in other samples and contexts to ensure its scalability.

Concerning some of our primary outcomes, we relied exclusively upon parental-report of child behavior for children under the age of 10. Because parents received the intervention and reported the outcomes, expectancy effects, social desirability, and increased awareness of screen use may have influenced the findings. Future work should consider incorporating objective measures of behavior (e.g., device-derived screen time). Other important outcome-related limitations include how our measure of screen time-related rules captured reported rule presence rather than enforcement, compliance, or effectiveness. Expanding this measure to include such aspects would elucidate the potential effects the intervention has on family functioning and rule-setting. Lastly, the sample was also characterized by a skewed gender distribution, with primarily boys and mothers participating, which may limit generalizability.

Strengths include the use of standardized measures, inclusion of both parent and older-child perspectives, and delivery within a routine clinical digital format. Future studies should incorporate objective screen-use data where feasible, child and clinician/teacher reports, measures of sleep, school functioning, parent–child conflict, emotional symptoms, gaming-related impairment, adherence, engagement, delivery fidelity, and longer follow-up.

### 4.2 Implications and future directions

P-FAME, as a brief, digitally delivered, parent-centered program, warrants further controlled evaluation in CAMHS. Future studies should test whether larger, more diverse samples and a comparison condition replicate the observed changes and should evaluate outcomes over longer follow-up periods. Digital refinements to P-FAME may benefit from leveraging personalization, real-time adaptive monitoring, multimodal data integration, and rigorous evaluation to enhance engagement and long-term behavioral impact. [36] For example, future work could examine whether parent guidance can be tailored to baseline screen-use patterns, parental stress, child developmental stage, ADHD status, family functioning, or rule-setting practices. Such features should be developed with attention to privacy, feasibility, and family burden.

Future evaluations should also assess implementation outcomes explicitly, including acceptability, engagement with intervention materials, adherence, barriers to participation, technical access, and fidelity of delivery. Digital and home-based formats may reduce access barriers, but their effectiveness and engagement can vary. [35] Finally, because the present study was conducted exclusively in a clinical CAMHS population, evaluation in school health, primary care, social-service, and preventive settings is needed before transferability beyond CAMHS can be inferred.

### 4.3 Conclusions

In this prospective single-group pre–post study, participation in P-FAME was associated with lower child screen time and parental stress at post-intervention, while family functioning did not change significantly. These findings are preliminary and do not establish efficacy or causality. P-FAME may be a useful candidate for further controlled research in clinical CAMHS settings; its effectiveness, mechanisms, implementation, and transferability to non-clinical settings remain to be established.

## Ethical statement

The study was approved by the Swedish Ethical Review Authority (DNR 2025-04039-02, 24 June 2025; DNR 2024-08667, 27 February 2025).

## Funding

The study was supported by the Y-ALF Swedish Government Research Grant (Ref. 89110; ECK), the Hains Foundation (ECK), the Swedish Freemasons Foundation for Children's Welfare (ECK), and the Centre for Clinical Research Region Dalarna (FA).

## Declaration of competing interest

The authors declare that they have no known competing financial interests or personal relationships that could have appeared to influence the work reported in this paper.
